# Almost an Earthquake: The Austrian Parliamentary Election of 2013

**DOI:** 10.1080/01402382.2014.895524

**Published:** 2014-04-28

**Authors:** Martin Dolezal, Eva Zeglovits

The Austrian election held on 29 September 2013 resulted in all-time lows for both major traditional parties, the SPÖ and ÖVP, but they nevertheless secured their combined majority by a tiny margin. Whereas the populist radical right FPÖ was supported by every fifth voter, its split-off, the BZÖ, lost parliamentary representation. The Greens achieved moderate gains and two new parties entered parliament: the populist Team Stronach and the liberal NEOS (Kritzinger *et al*. 2014). These results and a record low in turnout constitute important changes in Austrian politics, though the 2013 election falls short of having been a real ‘earthquake election’. Several features of the party system, especially in terms of coalition building, remained the same.^1^

## Background

The snap election of 2008 (Luther 2009; Müller 2009) had resulted in a renewed coalition of the Social Democrats (SPÖ) and the conservative People’s Party (ÖVP). Werner Faymann (SPÖ) became the new chancellor. From the start, this government was out of favour with public opinion, and commentators criticised its unwillingness to introduce substantial reforms.

As everywhere in Europe, the legislative period, which was extended from four to five years, was dominated by the global financial crisis and especially by troubles in the Eurozone. Though the government had to nationalise several banks, most prominently the Hypo-Alpe-Adria, Austria’s economic performance was above average. Many indicators, especially the rate of unemployment, continued to rank Austria considerably better than most other EU members. Apart from the economic crisis, politics centred on an endless series of corruption scandals. Many incidents originated in the early 2000s when the ÖVP had governed first with the Freedom Party (FPÖ) and later with the Alliance for the Future of Austria (BZÖ). Several top politicians were accused of illegally funding their parties or lining their own pockets. Because the SPÖ was also implicated in several cases, the Greens remained the only party without any scandal.

Apart from these problems, the five parties represented in parliament fared differently. Two party leaders, Faymann (SPÖ) and Heinz-Christian Strache (FPÖ), remained in power. The three other parties experienced changes, at times in a dramatic way: the BZÖ was severely hit by the sudden death of its founder Jörg Haider, who died in a car accident just two weeks after the election. Josef Bucher, a member of parliament since 2002, eventually became his successor. Since then, the party has had to fight for its survival. Apart from its last triumph in the 2009 Land election of Carinthia, where Haider had been Landeshauptmann (governor), the BZÖ lost all other elections and was no longer represented in any Land parliament. Furthermore, it also lost several members of its national parliamentary group to the FPÖ as well as to the newly founded Team Stronach (see below). In the ÖVP, Wilhelm Molterer, the vice-chancellor of the former government, who had provoked the snap election of 2008, was replaced by Josef Pröll. Due to health problems, Pröll resigned in 2011 and was succeeded by Michael Spindelegger, the foreign minister, as the new leader. The Greens also experienced a leadership change. After 11 years in office, Alexander van der Bellen was replaced by Eva Glawischnig immediately after the 2008 election.

In September 2012, a year before the (latest possible) day of the election, a new party was launched by the Austro-Canadian billionaire Frank Stronach. Born in 1932, Stronach had migrated to Canada in the 1950s where he founded Magna, a major international automotive supplier. Since the 1990s he had become a notable figure in Austria, not only as an entrepreneur but also, amongst other things, as president of the national soccer league. He also criticised the lack of political and economic reforms and finally presented his own party called Team Stronach. Just a few weeks later he managed to set up a parliamentary group thanks to five representatives of the BZÖ who changed their party affiliation, presumably because they did not expect re-election to parliament under the BZÖ label.

A further new party was launched only one month after Team Stronach, though with much less media hype. NEOS, its acronym standing for ‘The New Austria’, presented itself as a liberal, reform-minded group. The party leader, Matthias Strolz, as well as some others, originally came from the ÖVP’s business wing. In March 2013 NEOS announced an electoral alliance with the Liberal Forum, a party that had been represented in parliament in the 1990s but had become insignificant after the turn of the millennium.

While NEOS did not contest any sub-national election, Team Stronach immediately established itself as a competitive force in several Land elections. In three out of the four elections held in spring 2013 it won around 10 per cent of the votes (Carinthia, Lower Austria and Salzburg). Following internal conflicts, it failed to pass the threshold of representation in Tyrol. These successes were accompanied by setbacks for the FPÖ. Although Stronach did not pursue the issue of migration, he shares the populist radical right’s Euroscepticism and antipathy for the ‘political elites’ and therefore was seen as a major threat to the FPÖ in the upcoming election. The second major winner of Land elections was the Greens. After joining coalitions in Tyrol, Salzburg and Carinthia, they were represented in five out of nine Land governments in mid-2013, which was their best outcome yet (Dolezal 2014).

New parties face few constraints from the electoral system that employs a 4 per cent threshold. Reforms introduced in 2013 give more weight to preference votes. More fundamental were new rules for campaign finance, intended to provide more transparency and restrict party campaign spending. Accordingly, each party is limited to €7 million campaign spending in the period between the *Stichtag* (a date that is relevant for several aspects in electoral administration) and Election Day, specifically between 9 July and 29 September 2013. In previous elections both major parties had spent considerably more (Sickinger 2013).

## The Campaign

Apart from the new spending regulations, campaign length is not legally determined. All parties celebrated official kick-off meetings but, as usual, these events took place quite late to demonstrate the parties’ commitment to a short (and cheap) campaign. If one takes the presentation of the parties’ electoral manifestos and the first waves of campaign posters as indicators, however, the hot phase of the campaign started in mid-August, about six weeks before Election Day.

Traditional means of advertising such as newspaper ads and posters still characterise Austrian campaigns. TV spots, by contrast, are less important than in most other countries because parties are not allowed to broadcast them on public television (ORF), and private channels were not used much in 2013. From July to September the six parties represented in parliament plus NEOS and the tiny communist party (KPÖ) spent 88.9 per cent of their budget on adverts in newspapers and posters, and only 7.9 per cent on ‘modern’ means of advertising (TV, radio, cinema), and 3.3 per cent for adverts in online media.^2^

As expected, Stronach demonstrated his enormous financial superiority. His party alone accounted for one third of the overall spending of €32.4 million. Whether the parties obeyed the new spending limit remains an open question, as reports on their finances will not be published before September 2014. However, especially Team Stronach obviously far exceeded the limit. During the campaign the parties accused each other of illegally funding their campaigns. Especially the SPÖ, which had planned to use state subsidies assigned to its parliamentary group, was heavily criticised.

While traditional means of communication dominated, online campaigning also gained some importance. In terms of the number of fans on Facebook, the most widespread social medium in Austria, Team Stronach and NEOS were more successful than the established parties. FPÖ leader Strache, however, was by far the most visible top candidate. The prevalence of Facebook is also demonstrated by the rank-and-file candidates’ use of the internet: while only 6.6 per cent had a personal website and 16.1 per cent used Twitter, no less than 50.4 per cent had their own Facebook account.^3^ The voters responded to these efforts: more than half of those who use social media read something about the election on these platforms during the campaign.^4^

While TV spots are not important features of Austrian campaigns, televised debates are extremely popular and widely regarded as the most important campaign events (Plasser and Lengauer 2010). Since 1994 when the current practice was introduced, the top candidates of all parties represented in parliament confront each other in pair-wise debates on public television. With six parties represented, including Team Stronach (which had parliamentary representation due to defections from the BZÖ) but not NEOS, this led to a new record of 15 debates, plus a final round table. As a result of additional shows on ORF, plus similar programmes from private stations (ATV and PULS 4), interested viewers could see the top candidates almost daily on prime-time television in the final weeks of the campaign. The ORF debates were watched on average by 715,000 viewers, thus by more than 10 per cent of the electorate.

Especially the pair-wise debates gave the candidates a platform to present their policy positions but a lot of media attention was primarily focussed on their performance. According to surveys published in the daily *Kronen Zeitung*, Chancellor Faymann (SPÖ) ‘won’ all his five confrontations. Glawischnig (Greens), the only female top candidate in 2013, came second. Stronach, by contrast, was seen as the clear loser of the debates. His appearances were widely criticised, as he was not willing – perhaps also not able – to engage in discussions on policies. He rather accused his opponents, as well as the hosts, of lacking any economic knowledge and personal experience. In another TV show he proposed introducing the death penalty for ‘professional killers’, which caused public outrage and was repudiated by several leading members of his own party.

## Campaign Issues

The three most salient issues addressed by parties in the campaign via press releases or ads in newspapers were the economy, welfare and corruption.^5^ Differences between the parties were remarkable. The government parties mainly focussed on the economy and welfare. The SPÖ’s campaign in particular centred on unemployment and pension issues and thus targeted core voters, while Chancellor Faymann stressed Austria’s comparatively good economic performance. The ÖVP had a less focussed agenda. Nevertheless, economic issues were most salient also in the ÖVP campaign. The ÖVP strategy was to present a new scenario for the country’s future, with Spindelegger as the new chancellor.

All opposition parties made corruption the number one issue in their press releases; otherwise, the issue agendas were quite different. While the Greens were most focussed on corruption and occasionally added environmental issues, Team Stronach addressed welfare and economic issues, building on Stronach’s successful business career. Besides corruption the FPÖ focussed on welfare. Notably, immigration issues did not figure as the FPÖ’s most salient issues in its attempts to reach out to the media. However, in communications directed at the voters the FPÖ relied on its successful anti-immigration agenda. The BZÖ emphasised economic issues such as tax cuts and tried to position itself as a right-liberal party in an attempt to become more distinguishable from the FPÖ. NEOS differed from the other parties in that its campaign was least focussed on issues but rather concentrated on abstract topics, such as ‘courage for change’. However, whenever the party did address issues, the welfare state’s generational fairness and educational issues were mentioned. The NEOS campaign was thus primarily targeted at young and highly educated voters.

Interestingly, the issue agenda of the parties was only partly reflected by the media and the voters. In the media, corruption was the most salient issue, and thus even more salient than in the parties’ campaigns. Education became topical when the school year started in September. Here the main line of conflict was between the two government parties, with the SPÖ favouring a comprehensive school system, while the ÖVP defended differentiation at the age of 10.

Voters rather stuck to their usual issue preferences: the economy, welfare and education were most salient. Inter-party differences were remarkable and reflected the parties’ traditional core: welfare was most important for SPÖ voters, the economy for ÖVP voters, immigration for FPÖ voters, the environment plus education and culture for Green voters. Corruption was not a major concern of voters. Voters responded to the Greens’ track record and ranked them as the most competent party to fight corruption. Otherwise the voters tended to stick to traditional party competence ascriptions. This is most visible for the FPÖ: while it did not particularly focus on immigrant issues in the campaign, the FPÖ voters’ main concern remained immigration.

Notably, the EU or the euro crisis was hardly addressed at all. As there were no decisions taken in the EU (perhaps due to the general elections in Germany only one week before the Austrian ones), the subject was not prominently addressed by any party.

## The Results

Austria experienced an all-time low in turnout (74.9 per cent, i.e. 3.9 per cent down from 2008; see Table 1). Though still relatively high compared to other European countries, this drop nevertheless indicated important changes in voting behaviour. Turnout was especially low among the less religious, among naturalised migrants and among the young, thus among voters not connected to traditional cleavages such as class or religion (Kritzinger *et al.* 2013). In addition, after a long series of corruption scandals, increased political cynicism contributed to the decline in participation.

**TABLE 1:** ELECTIONS TO THE AUSTRIAN NATIONAL COUNCIL (29 SEPTEMBER 2013)

	**2013**			**2008**		
	**Seats (*N*)**	**Votes (000s)**	**Votes (%)**	**Seats (*N*)**	**Votes (000s)**	**Votes (%)**
Sozialdemokratische Partei Österreichs (SPÖ)	52	1,259	26.8	57	1,430	29.3
Österreichische Volkspartei (ÖVP)	47	1,126	24.0	51	1,270	26.0
Freiheitliche Partei Österreichs (FPÖ)	40	962	20.5	34	857	17.5
BZÖ – Liste Josef Bucher (BZÖ)^1^	0	166	3.5	21	523	10.7
Die Grünen – Die Grüne Alternative (GRÜNE)	24	583	12.4	20	510	10.4
Team Frank Stronach (FRANK)	11	269	5.7	–	–	–
NEOS Das Neue Österreich und Liberales Forum (NEOS)^2^	9	233	5.0	0	102	2.1
Bürgerforum Österreich Liste Fritz Dinkhauser (FRITZ)	–	–	–	0	86	1.8
Kommunistische Partei Österreichs (KPÖ)	0	48	1.0	0	37	0.8
Others	0	48	1.0	0	72	1.5
Total	183	4,693	100%	183	4,887	100%
Turnout (%)			74.9			78.8

^1^ In 2008: ‘BZÖ – Liste Jörg Haider (BZÖ)’.

^2^ In 2008 the Liberal Forum contested the election on its own. NEOS, founded in 2012, formed an electoral alliance with the Liberals. *Source*: Federal Ministry of the Interior.

**FIGURE 1:**
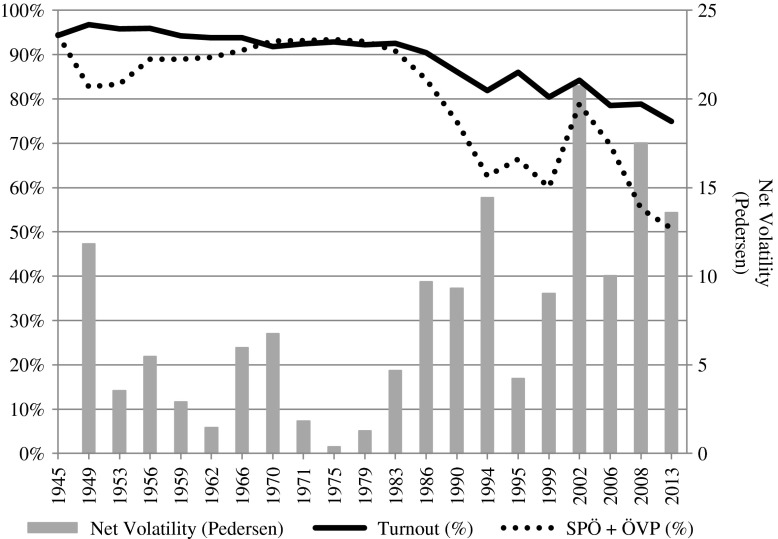
AUSTRIA’S POST-WAR ELECTORAL HISTORY: TURNOUT, STRENGTH OF MAJOR PARTIES (SPÖ AND ÖVP) AND NET VOLATILITY *Note*: The Pedersen index (Pedersen 1979) is based on the vote share of all parties with at least one per cent of the vote in a given election plus all parties that have won at least one seat since 1945. *Source*: Federal Ministry of the Interior.

The two traditional major parties finished at new record lows: the SPÖ won 26.8 per cent of the vote (–2.5), the ÖVP 24.0 per cent (–2.0). Their combined vote share thus dropped to 50.8 per cent (see Figure 1). The FPÖ, by contrast, increased its share by 3 points to 20.5 per cent and continued its recovery after its severe defeat in 2002 when it was in government (Luther 2003). In contrast, the FPÖ split-off, BZÖ, lost 7.2 points and all of its seats. Immediately after the election, party leader Bucher resigned. The Greens gained 2 points and attained their best result ever (12.4 per cent). However, neither the Greens themselves nor the media regarded them as clear winners, as opinion polls had forecast about 15 per cent and the SPÖ–ÖVP majority indicated that the Greens’ strategic goal of government participation could not be achieved.

The new parties fared differently: Team Stronach secured 5.7 per cent, which was widely interpreted as a disappointing result. It was a lower share than in the Land elections of 2013 and far behind original ambitions and predictions. NEOS, by contrast, was a clear winner as its unexpected share of 5.0 per cent secured parliamentary representation. Most polls had not seen it crossing the threshold until the very last days of the campaign. Given its relatively small budget and limited media presence, in particular on TV, this success was even more surprising.

From a long-term perspective, the 2013 election shows several features of a continued secular change in Austria’s political landscape. The decline of turnout and traditional parties, the strength of the populist radical right as well as higher levels of volatility than in earlier decades are the most important indicators (Figure 1). Some of the old cleavages, especially social class and religion, still have explanatory power on the individual level but only a minority of the electorate nowadays belongs to their defining groups (see Kritzinger *et al.* 2013).

## Government Formation

The electoral result produced three alternatives that had some political viability: the first being a remake of the grand coalition as the only politically viable two-party majority coalition. Together, the SPÖ and FPÖ would have controlled a razor-thin majority in parliament (92 out of 183 seats) but this option had been constantly ruled out by the Social Democrats since the FPÖ’s right turn in the mid-1980s. As the ÖVP could no longer form a majority coalition with the FPÖ its bargaining power was severely reduced.

Given the widespread criticism of the grand coalition two further alternatives were discussed. Some leading journalists argued for a coalition of SPÖ and ÖVP with the Greens and/or NEOS. But this idea was welcomed only by representatives of the latter. Another option was a three-party coalition of the centre-right, including the ÖVP, FPÖ and Team Stronach. However, the likelihood of this coalition immediately vanished as Team Stronach was hit by severe internal struggles which culminated in several Land-level leaders being removed or expelled from the party and Land parties dissolving themselves. Apparently dissatisfied with the election result, after a few months Stronach announced his intention to leave politics.

On 9 October President Heinz Fischer appointed Faymann, the leader of the strongest party, to form a new government. He also indicated sympathy for another grand coalition. The ÖVP accepted Faymann’s invitation for negotiations five days later but explicitly called them an ‘open-ended process’. Negotiations lasted for more than two months, during which time only little information was leaked to the public. The media reported substantial ideological gaps between the parties, in particular regarding pensions, education and taxes. Nevertheless, on 12 December both parties announced having reached a compromise and four days later the new government was sworn in.

The division of portfolios between the two parties remained roughly the same. While most of the SPÖ ministers remained in office, the ÖVP exchanged about half of its team, with Vice-Chancellor Spindelegger moving from Foreign Affairs to Finance.

## Conclusion

The 2013 election allows for different interpretations: while both traditional major parties experienced new record lows, they nevertheless won a combined majority and thus were able to continue with a ‘grand coalition’ government. The right-wing populist FPÖ was supported by every fifth voter; however, its offspring, the BZÖ, lost parliamentary representation. Team Stronach legitimised its status as a parliamentary party but its result was far behind original expectations. And even the Greens did not celebrate their new record high, as the actual result fell short of their hopes.

One week after the German election that had resulted in a severe defeat for the liberal FDP, its ideological sibling NEOS was the only clear winner in the Austrian elections. Whether this party will become a stable factor in Austrian politics and put an end to the absence of a liberal party in parliament remains to be seen. Also the fate of Team Stronach remains open. Compared to the first showing of other business tycoons in politics (e.g. Italy’s Silvio Berlusconi and more recently Andrej Babis in the Czech Republic) Stronach’s first national election almost ended in disaster.

## Notes

1. Other recent reports in this series include: Dinas and Rori (2013), Fenech (2013) and Garzia (2013).

2. Source: Focus Media Research. The aggregation was done by the authors.

3. These figures are based on a sample of 1,000 candidates (out of a total of 3,946).

4. This figure is based on one of the AUTNES post-election voter surveys.

5. Based on data collected by the Austrian National Election Study AUTNES (http://www.autnes.at/).
